# Spatial factors influencing the pain-ameliorating effect of CT-optimal touch: a comparative study for modulating temporal summation of second pain

**DOI:** 10.1038/s41598-024-52354-3

**Published:** 2024-02-01

**Authors:** Larissa L. Meijer, Wouter Baars, H. Chris Dijkerman, Carla Ruis, Maarten J. van der Smagt

**Affiliations:** 1 Utrecht University, Heidelberglaan 1, 3584 CS Utrecht, The Netherlands; 2 University Medical Centre Utrecht, Utrecht, The Netherlands

**Keywords:** Neurophysiology, Insula, Sensory processing

## Abstract

Recent studies show that CT-optimal touch, gentle slow stroking of the skin, can reduce pain. However, much is unknown regarding the factors influencing its pain-ameliorating effect, such as tactile attention and touch application site. The current study investigates in 36 healthy individuals, whether CT-optimal touch can reduce temporal summation of second pain (TSSP) compared to CT non-optimal touch and tapping the skin. TSSP refers to activation of the C-nociceptors; by stimulating these fibers a burning and/or tingling sensation can be elicited. All participants underwent three conditions on both the contralateral and ipsilateral side of pain induction. The results show that tapping the skin did not reduce TSSP, meaning that pain reduction through touch cannot be explained by tactile attention effects. CT non-optimal touch only reduced TSSP when applied on the ipsilateral side. Importantly, CT-optimal touch effectively reduced TSSP when applied on the contralateral or ipsilateral side. Furthermore, CT-optimal touch was more effective in reducing TSSP compared to CT non-optimal touch and Tapping. This study shows that that CT-optimal touch can reduce TSSP and this effect appears to be independent of touch application site, which is highly relevant for implementing CT-optimal touch as a treatment.

## Introduction

Touch is important to discriminate, localize and identify stimuli that contact our skin. Beside this more discriminative role, touch also plays an important role in interacting with one another and is thereby vital for social bonding^1^. A particular type of touch appears to be involved in these more affective and social aspects namely, affective touch or CT-optimal touch. CT-optimal touch refers to activation of the C-tactile system by gently stroking the skin at an optimal velocity of 3 cm/s with a range of 1–10 cm/s^2^.

Recent studies show that besides mediating tactile pleasantness, CT-optimal touch can reduce acute and chronic pain experience^3–8^. Based on the model of^9^ activation of the CT-system can influence pain perception through two mechanisms. The first one is a bottom-up peripheral mechanism which inhibits pain signals at the level of the dorsal horn of the spinal cord. The second one is a top-down regulatory system within the insula, with pain signals being down-regulated resulting in lower pain levels. These studies show that CT-optimal touch might be a promising addition to current pain treatments. However, even though these studies show promising results, much is still unknown regarding the factors influencing the pain-ameliorating properties of CT-optimal touch.

One of these factors is tactile attention. When touch is applied on the skin, our attention is almost automatically drawn towards that stimulus^10^. Previous studies show that attention can serve as a pain distractor^11^. However, these previous studies mostly used visual, auditory or cognitive tasks to distract from perceived pain. Whether tactile attention can also be used as a pain distractor therefore remains unknown. As the neurophysiological mechanisms underlying CT-optimal touch are not fully understood yet, it could also be that the previously found effects of CT-optimal touch on pain experience (partially) rely on attentional effects^8^. Studies into the effect of CT-optimal touch on pain often use a faster touch as a control condition, also referred to as CT non-optimal touch. Even though CT-optimal touch is significantly more effective than CT non-optimal touch, CT non-optimal touch can also reduce pain experience to some extent^5–8^. This might also be related to spatial tactile attention. Therefore, in the current study an extra stimulus condition (tapping instead of stroking) is used to control for this possible attentional effect.

Another factor influencing the pain-ameliorating properties of CT-optimal touch might be the touch application site. Most studies into the effect of CT-optimal touch on acute pain applied touch on the same body part as where pain was induced^5,6,12^. There are only two studies where touch application and nociceptive stimulation were spatially distinct^8,13^. In addition, there are two studies showing that CT-optimal touch effectively reduces chronic pain experience when applied on the same body part^4,7^. Another study into CT-optimal touch and chronic pain experience did not report the exact body part affected by the chronic pain condition and it is therefore unclear whether touch application was, for instance, contralateral or ipsilateral to the pain location^3^. Taken together, current studies show that CT-optimal touch effectively reduces pain experience either ipsilateral or contralateral to the pain location. However, none of these studies actually *compared* ipsilateral to contralateral touch application. Therefore, it is unknown if there is any difference in effectiveness. As CT-optimal touch can interact with pain processing on both a peripheral and a central level of the nervous system^9^, one might argue that when touch is provided ipsilaterally, the peripheral system can inhibit the C-nociceptors at the level of the spinal cord and thereby prevent further pain processing. When touch is applied ipsilaterally the peripheral system is activated first, CT-optimal touch already interferes with pain processing on the level of the spinal cord and top-down regulation might not be activated or to a lesser extent. We therefore hypothesize that CT-optimal touch is more effective when applied on the ipsilateral side.

In order to study this, we adapted a stimulation protocol from Fidanza et al.^4^ which uses repetitive heat pulses to induce Temporal Summation of Second Pain (TSSP) also referred to as wind-up pain. This paradigm activates C-nociceptors,by repetitively stimulating these fibers a burning and/or tingling sensation can be elicited^14^. This is linked to central neuronal sensitization, a process related to chronic pain. Therefore, inducing TSSP in healthy individuals can serve as a model for chronic pain conditions^14^. The study of Fidanza et al.^4^ shows that CT-optimal touch can reduce TSSP compared to a no touch condition and very slow (0.3 cm/s) touch. However, in this study CT-optimal touch was applied only ipsilaterally to nociceptive stimulation. Therefore, in the current study participants will undergo three types of tactile stimulation while TSSP is induced namely, CT-optimal touch (3 cm/s), CT non-optimal touch (18 cm/s) and a Tapping condition. A velocity of 18 cm/s has proven to be an effective control condition for CT-optimal touch and is quite natural to apply^7,8,15^. The Tapping condition is adapted from McIntyre et al.^16^ and will be used as a control condition for spatial tactile attention. All types of touch will be applied both ipsilaterally and contralaterally to nociceptive stimulation. We hypothesize that CT-optimal touch will effectively reduce TSSP compared to CT non-optimal touch and Tapping and that this effect will be larger when CT-optimal touch is applied on the ipsilateral side compared to the contralateral side^7^.

Furthermore, similar to Fidanza et al.^4^ we also look into the relationship between body awareness and the pain-relieving effect of CT-optimal touch. Previous literature shows that the ability to detect internal states of the body i.e. body awareness, is related to pain perception^17^ as well as to CT-optimal touch^18^. Even more so, people suffering from chronic pain also report higher levels of body awareness^19^, and therapies targeting body awareness appear to reduce some forms of chronic pain^20^. As such, we expect that participants who report high levels of body awareness, as measured with the Body Perception Questionnaire Short Form (BPQ), might benefit more from the pain-ameliorating effect of CT-optimal touch as this is also an interoceptive modality^21^. In addition, they might also perceive touch as more pleasant as they might be more prone to these bodily sensations.

## Results

Before the start of the experiment participants’ baseline temperature was determined. Overall, the average baseline temperature was 49.01 °C (SD = 1.72). The participants also filled out the Body Perception Questionnaire (BPQ) Short Form as a measure for body awareness. The participants’ average total item score was 38.08 (SD = 10.12).

### Main effect tactile stimulation

**Figure 1:**
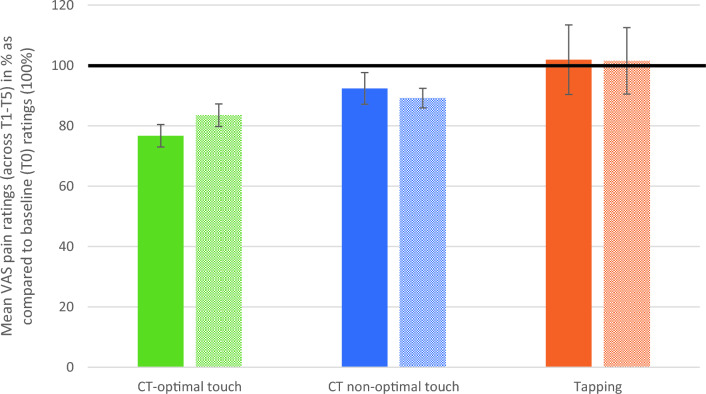
Mean VAS pain ratings (across T1–T5) in % as compared to baseline (T0) ratings (100%). Filled bars depict contralateral administration, textured bars ipsilateral administration. Error bars depict ± 1 standard error of the mean.

TSSP was measured with the VAS pain scale. Based on Fidanza et al.^4^ we calculated an average VAS pain score for every condition (T1-T5). As we used a baseline trial (a trial without touch) instead of a ‘no touch’ condition as used by Fidanza et al.^4^, we compared the VAS pain score on T0 (baseline) with the scores reported during the intervention (see Table S1 and Fig. 1). This was done by using a time (baseline x intervention) × condition (CT-optimal touch × CT non-optimal touch × tapping) × site (contralateral × ipsilateral) repeated measures ANOVA. We found a significant main effect for time F(1,35) = 24.82, *p* =  < 0.001, partial η^2^ = 0.42 and a significant main effect for site F(1,35) = 4.72, *p* = 0.037, partial η^2^ = 0.12. There was no significant main effect for condition (p = 0.185). There was a significant interaction effect for time*condition (*p* = 0.006). There was no significant interaction for condition*site (*p* = 0.511), nor for the interaction time*condition*site (*p* = 0.525). Pairwise Bonferroni corrected comparison shows a significant difference between baseline and intervention for CT-optimal touch and CT non-optimal touch but not for Tapping (see Table 1).

**Table 1:** Mean difference baseline vs. intervention and p-value for every condition.

	Mean difference T0 vs. M (T1-T5)	*p*-value
CT-optimal touch	11.21	< .001
CT non-optimal touch	6.38	.015
Tapping	4.31	.354

In addition, in order to analyze the difference between baseline vs. intervention within a single condition a Simple Main Effects analysis was done (see Table 2). We found a significant effect for CT-optimal touch on both the contralateral and ipsilateral side. For CT non-optimal touch there was a significant effect on the ipsilateral side but not on the contralateral side. There was no significant effect for Tapping, meaning that tapping the skin did not significantly reduce pain experience (see Fig. 1).

**Table 2:** Results simple main effects analysis.

	F	*p*-value*
CT-optimal touch *contralateral*	32.39	< .006
CT-optimal touch *ipsilateral*	18.84	< .006
CT non-optimal touch *contralateral*	7.41	.060
CT non-optimal touch *ipsilateral*	14.10	< .006
Tapping *contralateral*	5.12	.180
Tapping *ipsilateral*	2.91	.582

*Bonferroni corrected *p*-value.

### Difference between conditions

In order to investigate whether there was a significant difference between conditions, we decided to use a condition (CT-optimal touch x CT non-optimal touch x tapping) x site (contralateral x ipsilateral) repeated measures ANOVA. Here, we used the mean difference scores for the VAS pain scale.

We found a significant main effect for condition F(2,70) = 6.28, *p* = 0.006, partial η^2^ = 0.15. There was no significant main effect for site (*p* = 0.421), nor for the interaction condition*site (*p* = 0.525). Pairwise Bonferroni corrected comparison shows a significant difference between CT-optimal touch and Tapping (*p* = 0.021) and between CT-optimal touch and CT non-optimal touch (*p* = 0.019). There was no significant difference between CT non-optimal touch and Tapping (*p* = 0.813).

### Pleasantness tactile stimulation

The pleasantness of tactile stimulation measured with the VAS pleasantness was analyzed with a repeated measures ANOVA. Data was normally distributed; sphericity was violated so Greenhouse–Geisser corrections were used. One participant did not report pleasantness ratings for CT non-optimal touch onthe contralateral side therefore N = 35. The VAS mean pleasantness ratings are depicted in Table S1.

The repeated measures ANOVA showed a significant main effect for condition F(2,68) = 10.37, *p* < 0.001, partial η^2^ = 0.23. There was no significant main effect for site (*p* = 0.263), nor an interaction for condition * site (*p* = 0.217). Pairwise Bonferroni corrected comparisons shows that both CT-optimal touch and CT non-optimal touch were perceived as significantly more pleasant than Tapping (*p* = 0.002 and *p* = 0.007 respectively) There was no significant difference between CT-optimal touch and CT non-optimal touch (*p* = 1.00), meaning that both CT-optimal touch and CT non-optimal touch are perceived as more pleasant than Tapping, but did not differ from each other.

### Other analyses

As CT-optimal touch applied on the contralateral and ipsilateral side significantly reduced TSSP, a possible relationship between the VAS pain difference scores and perceived pleasantness was investigated by using a Spearman correlation (see Table 3). This shows that there is no significant correlation between CT-optimal touch and perceived pleasantness on the contralateral side. However, there was a significant correlation between CT-optimal touch and perceived pleasantness on the ipsilateral side. As CT non-optimal touch on the ipsilateral side also significantly reduced pain, we also investigated whether this ipsilateral effect was related to pleasantness. Spearman correlations showed no significant correlation between CT non-optimal touch (ipsilateral side) and pleasantness (see Table 3).

**Table 3:** Spearman correlations.

Variable	BPQ	CT non-optimal touch ipsilateral pain	CT optimal touch contralateral pain	CT optimal touch ipsilateral pain
BPQ	–	.27	−.03	.12
CT-non optimal touch contralateral pleasantness	−.01	–	–	–
CT-non optimal touch ipsilateral pleasantness	−.02	.22	–	–
CT optimal touch contralateral pleasantness	.09	–	.29	–
CT optimal touch ipsilateral pleasantness	−.14	–	–	.43**
Tapping contralateral pleasantness	−.05	–	–	–
Tapping ipsilateral pleasantness	−.06	–	–	–

*p < .05, **p < .01, ***p < .001.

In addition, a Spearman correlation was used to analyze if there is any relationship between the BPQ-scores and the VAS pain difference scores for CT-optimal touch (both sides) and CT non-optimal touch (ipsilateral side). Spearman correlations showed neither a significant correlation between the BPQ scores and CT-optimal touch (both sides) nor for CT non-optimal touch (ipsilateral side). Furthermore, to investigate whether there was a relationship between the BPQ and perceived pleasantness, for every condition a Spearman correlation was used. There was no correlation between the BPQ and perceived pleasantness for every condition (see Table 3.)

## Discussion

Previous research has shown that CT-optimal touch can reduce pain in healthy individuals^5,6,8^ and in chronic pain patients ^3,7^. In addition, the study of Fidanza et al.^4^ shows that CT-optimal touch can also reduce Temporal Summation of Second Pain (TSSP). This is interesting as it appears that TSSP induced in health individuals can serve as a model for chronic pain^14^. However, in the^4^ study touch was applied only to the same body part as where TSSP was induced. As CT-optimal touch appears to reduce chronic pain as well, it is necessary to investigate which factors contribute to this effect. Two of these factors might be spatial tactile attention and touch application site. As touch in general can also generate attention to the stimulus touching the skin, it could be that previously found effects of CT-optimal touch on pain rely mainly on attentional effects^8,11^. Therefore, in this study we investigated whether spatial tactile attention could influence the pain experience. Furthermore, touch was applied both ipsilateral and contralateral side to TSSP induction to investigate whether touch application site matters. In the current study we show that CT-optimal touch when applied either on the contralateral or ipsilateral side effectively reduces TSSP. For CT non-optimal touch this effect was only found for the ipsilateral side.

In addition, we show that tapping the skin, used as a form of directing tactile spatial attention, does not reduce TSSP. Therefore, the pain-ameliorating effect of CT-optimal and CT non-optimal touch cannot be explained by tactile attention, i.e. using touch as a distractor is not sufficient to reduce experienced pain. This is not in line with previous studies into the effect of attention on pain^11^. However, previous studies mostly used visual, auditory or cognitive tasks as a distractor. There are only a few studies in which a tactile condition was used as a pain distractor, most of which used a very different type of tactile distraction i.e. non-painful electrical stimulation^11^. One study used tactile vibration as a tactile distractor, which seems more in line with the tactile Tapping condition used in the current study^22^. Here, they found that tactile vibration did not significantly reduce pain experience, which is in line with our results.

Furthermore, we also looked at the difference in pain reduction between conditions. Here, we show that CT-optimal touch reduces TSSP compared to CT non-optimal touch and Tapping. Specifically, it appears that this effect is independent of touch application site. This is in line with previous studies showing that CT-optimal touch can reduce pain when applied on the contralateral side^8,13^ as well as on the ipsilateral side^4,7^. However, these previous studies did not compare the effectiveness between applying CT-optimal touch contralateral or ipsilateral to pain side. The present study shows that the pain-ameliorating effect of CT-optimal touch is independent of touch site. This is substantiated by previous research showing that CT-optimal touch can interact with pain processing on a peripheral and a central level of the nervous system^9^. Even though in this study pain was induced in healthy individuals, these results provide important insights for the implementation of CT-optimal touch as a treatment for chronic pain. Chronic pain often expresses as back pain or joint pain but visceral pain is also common^23,24^. These pain sides are sometimes difficult to reach through touch. As we show that the effectiveness of CT-optimal touch is independent of touch site, CT-optimal touch might also be effective for more internal pain syndromes and can be applied on a different body part than where pain is perceived.

We also looked at the perceived pleasantness of the touch conditions. We show that Tapping is perceived as less pleasant compared to CT-optimal touch and CT non-optimal touch. There was no difference between CT-optimal touch and CT non-optimal touch. This is not in line with previous research showing that CT-optimal touch is perceived as more pleasant than CT non-optimal touch^4,8^. However, in the study of von Mohr et al.^8^ pleasantness ratings were collected prior to pain stimulation. In our study pleasantness was reported at the end of each block, based on Fidanza et al.^4^. As pleasantness was measured after pain stimulation this might have influenced the perceived pleasantness. Fidanza et al.^4^ did find a difference in perceived pleasantness but used a very slow touch instead of a faster touch, it is therefore difficult to directly compare these results. Another explanation might be that with a velocity of 18 cm/s the CT-fibers were slightly activated as well. It is known from microneurography studies that the optimal velocity to activate the CT-fibers is 1–10 cm/s and that a velocity of > 10 cm/s only activates a handful of CT-fibers. However, how the CT-fibers react to precisely 18 cm/s has not been tested yet^25^.

In addition, previous research shows that touch can have a pain-relieving effect through pleasantness and top-down related analgesic effects^26^. As we in general did not find this relationship between perceived pleasantness and the effect of CT-optimal touch and CT non-optimal touch on pain, this pleasantness related analgesic effect cannot explain our results. This is in line with a case study of^7^ in which a patients with neuropathic pain did not perceive CT-optimal touch as pleasant but did report complete pain-amelioration.

As mentioned, CT non-optimal touch only effectively reduced pain when applied on the ipsilateral side. As outlined above, we are not sure whether CT non-optimal touch with a velocity of 18 cm/s activates the CT-fibers to some extent. However, as CT non-optimal touch on the contralateral side did not reduce TSSP, the effect found on the ipsilateral side might be explained by the Gate Control Theory^27^, which refers to a ‘gate’ in the spinal cord which can be closed to interfere with pain processing. Closing the ‘gate’ appears to be related to activation of the large myelinated Aβ-fibers. The Aβ-fibers can be activated by stroking or rubbing the painful body part at a relatively high velocity^1^. The Gate Control Theory therefore appears to be based on a peripheral mechanism which can only be activated when touch is applied on the painful body part. As we used a velocity of 18 cm/s for the CT non-optimal touch condition it is likely that the Aβ-fibers were activated, and thereby the ‘gate’ to interfere with pain processing. This is further substantiated by the observation that CT non-optimal touch applied on the contralateral side was ineffective.

In our study we also looked into the possible relationship between body awareness, the pain-relieving effect and pleasantness. We expected that participants who report high levels of body awareness might benefit more from the pain-ameliorating effect of CT-optimal touch^17,19,20^. However, we did not find any relationship between body awareness and the pain-relieving effect either for CT-optimal touch or CT non-optimal touch applied on the ipsilateral side. There was also no relationship between body awareness and perceived pleasantness for any condition. This shows that in this study body awareness did not influence the pain-relieving effect of touch or the perceived pleasantness.

This study is not without limitations. Firstly, contralateral and ipsilateral touch application were not counterbalanced. During the set-up of this study our goal was to counterbalance every condition, however we believed that full counterbalancing could potentially lead to an increased transfer of effects between conditions. The interaction between stimuli might result in unintended influence bleeding over from one condition to another, possibly obscuring the effects we aimed to study. Therefore, we decided to only counterbalance the touch conditions perfectly, but fix the stimulation side order. Based on current knowledge of the CT-system and the two mechanisms involved in its pain relieving effect^9^, we hypothesized that starting on the ipsilateral side would increase the chance of unintended bleeding over effects. This is because when CT-optimal touch is applied on the ipsilateral side it is likely that both the peripheral and top-down pain inhibiting mechanisms are activated. Therefore, we theorized that starting on the contralateral side would decrease the chance of activating the peripheral inhibitory system and thereby avoiding any bleeding over effects that have a peripheral origin. However, even though we did counterbalance between conditions, in future studies, it is recommended to counterbalance ipsi- and contralateral side as well, to avoid any unwanted order effects. In addition, it would be of interest to add neurophysiological measures such as EEG to measure the amplitude of the N1, N2 and P2 complexes as these are related to noxious processing and compare this when CT-optimal touch is applied on the ipsilateral or contralateral side^8^. Secondly, after the study some participants subjectively reported that when touch was applied on the ipsilateral side it sometimes appeared to synchronize with the TSSP induction. This caused a feeling of sensory overload due to which CT-optimal touch seemed to be overruled by the TSSP induction resulting in higher pain ratings compared to the contralateral side. This is important as this might have influenced the effectiveness of CT-optimal touch. In addition, this might also have influenced the perceived pleasantness. However, as there was no difference between perceived pleasantness on the contralateral and ipsilateral side it appears unlikely that the reported synchronization also influenced the perceived pleasantness. Lastly, pain is a complex somatosensory sensation as we only measured pain intensity with a VAS scale this cannot fully cover all facets of pain perception. Moreover, as in the current study pain was induced in healthy individuals all statements regarding chronic pain are preliminary.

To conclude, we show that CT-optimal touch can reduce pain compared to CT non-optimal touch and Tapping. Furthermore, this study shows that spatial tactile attention is ineffective in reducing temporal summation of second pain. Therefore, tactile attention cannot explain the effect of touch on pain perception. Interestingly, the pain-ameliorating effect of CT-optimal touch appears independent of touch application site. Therefore, it seems that CT-optimal touch can also be applied on a different bodily location than on the pain location itself, which is highly relevant for implementing CT-optimal touch as a treatment.

## Methods

### Participants

A total of 38 healthy volunteers participated in this study between 01/06/2023 and 04/08/2023. The participants sample consisted of 20 males, 16 females and 2 participants whose gender was unspecified. The age range of the participants was between 18 and 32 (mean ± SD = 24.9 ± 3.3). Out of the 38 participants, 35 were right-handed. Any health conditions that could alter pain or tactile perception were considered as exclusion criteria. Two participants were excluded due to language barriers that hindered their understanding of the instructions. To maintain perfect counterbalancing these participants were replaced, as such 38 volunteers participated but data of 36 was used. Prior to participation, all individuals provided written informed consent, and their identity was anonymized throughout the study. The study protocol (Protocol Number: 23-0147) was reviewed and approved by the local faculty ethical review board at the Faculty of Social and Behavioral Sciences, Utrecht University. This research adhered to the ethical principles outlined in the WMA Declaration of Helsinki 2013.

### Thermal stimulation

TSSP was induced using a TSA-II Neuro Sensory Analyzer (Medoc Ltd., Advanced Medical Systems, Ramat Yishai, Israel). A thermode (30 × 30 mm) was positioned on the ventral side of the participant's left wrist to deliver trains of 6 heat pulses at 0.33 Hz. The stimulation method, derived from Staud et al.^14^, involved continuous-contact heat application. Each pulse encompassed an ascending and descending temperature change of 8 °C/s, with a complete cycle lasting 3 s. Individual target temperatures were adjusted based on heat pain sensitivity, aiming for maximal thermal TSSP ratings of 45 ± 10 after 6 heat pulses at 0.33 Hz. Following each stimulus train, pain ratings were collected using a Computerized Visual Analogue Scale developed with the Gorilla Experiment Builder (www.gorilla.sc).

### Tactile stimulation

In the present study, participants underwent three distinct conditions of tactile stimulation simultaneously with the induction of temporal summation of second pain (TSSP). These tactile stimulation conditions were CT-optimal touch at a velocity of 3 cm/s, CT non-optimal touch at a velocity of 18 cm/s, and a Tapping condition. The order of tactile stimulation was perfectly counterbalanced between participants. All forms of tactile stimulation were applied both contralateral and ipsilateral to the nociceptive stimulation side, on the dorsal part of the participants' forearm. The CT-optimal and CT non-optimal touches were manually administered with a soft brush in a proximal-to-distal direction by a trained experimenter. To ensure that the correct velocity was used throughout the experiment a metronome was used through an earplug. The Tapping condition was administered as a continuous series of taps with random intervals of ~ 0.1 to 2.5 s with a soft rubber tip of a pen, this method was adapted from McIntyre et al.^16^ and serves as a metric for evaluating spatial tactile attention.

### Questionnaires

Pain perception was measured with a Visual Analog Scale (VAS) ranging from 0 – 100, in which 0 represented ‘comfortable/unpainful’ and 100 ‘uncomfortable/painful’. Similarly, the VAS for Pleasantness assessed tactile stimulus pleasantness on a scale from 0 ‘unpleasant’ to 100 ‘pleasant’". To measure bodily awareness the Body Perception Questionnaire (BPQ-Short Form) was used^28^. The BPQ-Short Form contains 12 questions regarding body awareness which are scored on a 5-point (1 never—5 always) Likert scale. A total score between 12 and 60 can be obtained. Values at the high end of the scale reflect hypersensitivity and values at the low scale reflect hyposensitivity. In the present sample Cronbach’s α was 0.90.To rule out any possible order effects, half of the participants filled in the BPQ before the start of the experiment, while the other half filled in the BPQ at the end of the experiment.

### Procedure

Upon arrival participants received and read the information letter. After addressing any questions arising from the information letter, participants provided written informed consent.

**Figure 2:**
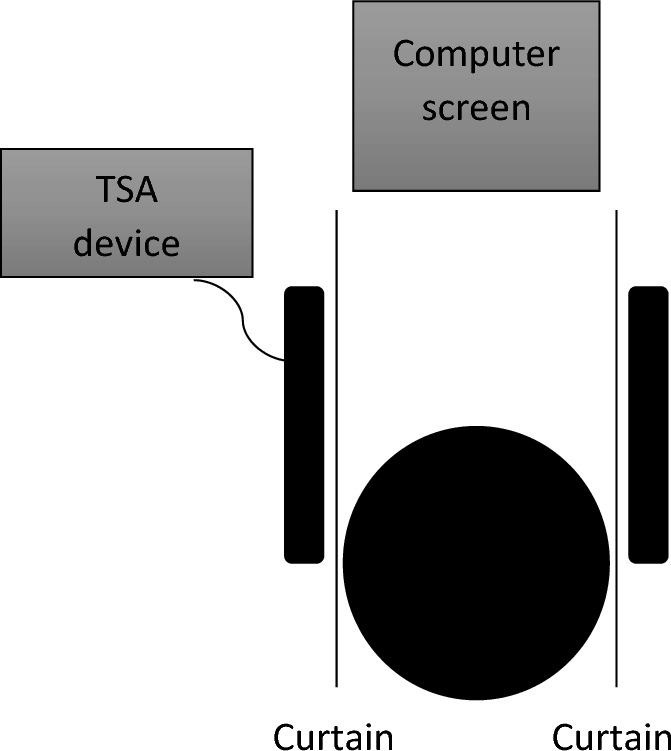
Schematic overview of experimental set-up.

The participants were prevented from seeing the stimulated skin area using a curtain (see Fig. 2). This was done to reduce visual distraction from the TSA device and to minimize social and top-down factors linked to touch administration.

Before the experiment started, the experimenter marked an area of 18 cm on the dorsal side of the participants' forearms to ensure consistent manipulations. A preliminary demographic questionnaire containing age, gender and handedness was administered through a computer interface. In addition, the participant filled in the Body Perception Questionnaire (BPQ) either at the start of the experiment or at the end. After this, the correct temperature for the heat pulses was determined by starting at a temperature of 45° Celsius and increasing or temperature until participants reported 45 ± 10 on the VAS pain scale with a maximum temperature of 50.5° Celsius. This was based on the protocol of^4^.

**Figure 3:**
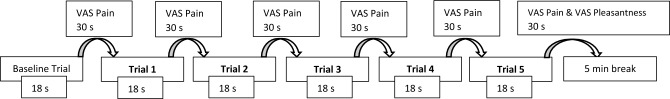
Schematic representation of a touch condition: during each condition 6 trials consisting of 6 heat pulses were delivered with a thermode on the ventral side of the participants left wrist. This thermode was calibrated before the start of the experiment per participant to elicit a tingling and burning sensation rated 45 ± 10 on the VAS pain scale. All three types of touch were administered continuously from trial 1 and onwards. One condition consists of one block on the contralateral arm and one on the ipsilateral arm.

For a visualization of the study procedure see Fig. 3. Before the start of every condition a baseline trial without any touch was conducted to establish a reference point for individual pain thresholds. Hereafter, touch was administered continuously over five trials in which TSSP was also conducted. Following each trial, participants filled in the VAS pain scale. Furthermore, participants rated the pleasantness of the touch at the conclusion of each condition. Intervals of 5 min were allocated as breaks after completing each condition. Upon completion of the experiment participants were compensated in the form of course credits (as part of the Psychology Bachelor curriculum at Utrecht University students need to participate in research to obtain course credits) or monetary remuneration. Any remaining queries were addressed before concluding the session.

### Statistical analysis

All data was processed using Microsoft Excel (version 2208) and analyzed with JASP (version 18.01). An apriori power calculation for a repeated measures ANOVA with expected power (0.80), medium effect size Cohen’s F (0.25) and alpha (0.05) recommend a sample size of 36. In order to analyze if there is any difference in pain perception between baseline and the different timepoints for every condition, a repeated measures ANOVA was used with time (baseline x intervention), condition (CT-optimal touch x CT non-optimal touch x tapping) and site (contralateral x ipsilateral) as within-subject factors. The VAS pain scores on T0 were used as the baseline factor. The intervention factor consisted of the average scores of T1–T5, as done by^4^. Data was checked for normality by using the Shapiro–Wilk test, visually inspecting the Q–Q plots and Skewness and Kurtosis were reported; more detailed information can be found in the Supplementary Table S1. Three out of twelve variables were not normally distributed according to the Shapiro–Wilk test. However, Skewness and Kurtosis were within an acceptable range. Sphericity was violated for one of the factors, therefore the Greenhouse—Geisser correction was used. In addition to the ANOVA, a simple main effect analysis was done to investigate a difference in pain perception within each condition.

To further analyze the difference in pain perception between conditions and touch site a repeated measures ANOVA was used. Here, condition (CT-optimal touch x CT non-optimal touch x tapping) and site (contralateral x ipsilateral) were used as within-subject factors. The dependent variable was the VAS pain difference score calculated by subtracting the average of T1–T5 from T0 (baseline). Four out of six variables were not normally distributed according to the Shapiro–Wilk test. However, when visually inspecting the Q–Q plots and histograms, two of these four variables appeared normally distributed and were within acceptable Skewness and Kurtosis range. As only two out of six variables were not normally distributed, sample size was relatively large (N = 36), a non-parametric alternative for a factorial ANOVA is not readily available, and it has been shown that Type 1 error and power of the F-statistic are not necessarily altered by violation of normality^29^, we decided that it was permitted to use a parametric test. Sphericity was violated so Greenhouse–Geisser corrections were used.

To analyze the difference in perceived pleasantness of touch, a condition (CT-optimal touch x CT non-optimal touch x tapping) x site (contralateral × ipsilateral) repeated measures ANOVA was used. The dependent variable was the perceived pleasantness measures with the VAS. Here only one variable was not normally distributed according to the Shapiro–Wilk test, but Skewness and Kurtosis were within an acceptable range. Sphericity was violated so Greenhouse–Geisser corrections were used.

Lastly, to analyze whether there is a relationship between the two dependent variables; pain perception and perceived pleasantness a Spearman correlation was used. Furthermore, a Spearman correlation was also used to analyze a possible relationship between the BPQ scores and pain perception as well as with perceived pleasantness.

### Supplementary Information

Supplementary Information.

## Data Availability

The datasets generated during and/or analysed during the current study are available in the YODA repository, DOI: 10.24416/UU01-PPZYF8.
