# Supplementary material for: Spatial factors influencing the pain-ameliorating effect of CT-optimal touch: a comparative study for modulating temporal summation of second pain
Source: Sci Rep. 2024 Feb 1;14:2626. doi: 10.1038/s41598-024-52354-3 (PMC10831064; doi:10.1038/s41598-024-52354-3)
Supplement: Supplementary file 1 — Supplementary Information. [file 41598_2024_52354_MOESM1_ESM.docx]

Supplementary Material File

**Spatial factors influencing the pain-ameliorating effect of CT-Optimal touch: a comparative study for modulating Temporal Summation of Second Pain**

Larissa L. Meijer, Wouter Baars, H. Chris Dijkerman , Carla Ruis and Maarten J. van der Smagt

Table S1. Descriptive statistics.

|  | Mean (SD) | Skewness | Kurtosis | Shapiro-Wilk  p-value |
| --- | --- | --- | --- | --- |
| *VAS pain scores* |  | | | |
| CT non-optimal touch contra T0 | 50.83 (16.10) | -1.12 | 1.22 | .91  .008* |
| CT non-optimal contra T1-T5 | 44.66 (16.55) | -.63 | -.63 | .93  .019* |
| CT non-optimal touch ipsi T0 | 54.47 (14.17) | -.70 | 1.38 | .94  .063 |
| CT non-optimal ipsi T1-T5 | 47.89 (14.10) | -.70 | .18 | .96  .168 |
| CT optimal touch contra T0 | 55.78 (14.90) | .04 | .73 | .97  .335 |
| CT optimal contra T1-T5 | 43.23 (17.71) | -.19 | -.65 | .98  .596 |
| CT optimal touch ipsi T0 | 55.61 (16.24) | -.58 | 1.26 | .95  .100 |
| CT optimal ipsi T1-T5 | 45.74 (16.77) | -.26 | -.56 | .98  .730 |
| Tapping contra T0 | 53.61 (14.48) | -.81 | 2.21 | .94  .037* |
| Tapping contra T1-T5 | 48.68 (14.61) | .07 | -.69 | .98  .772 |
| Tapping ipsi T0 | 55.25 (15.59) | -.54 | .72 | .95  .141 |
| Tapping ipsi T1-T5 | 51.57 (15.50) | -.04 | .53 | .97  .410 |
|  |  |  |  |  |
| *VAS pain difference score T0 – M(T1-T5)* |  | | | |
| CT non-optimal contra diff score | 6.18 (13.62) | 1.44 | 2.62 | .89  .002* |
| CT non-optimal ipsi diff score | 6.58 (10.51) | .87 | 3.57 | .94  .041* |
| CT optimal contra diff score | 12.54 (13.23) | 1.30 | 2.90 | .91  .008* |
| CT optimal ipsi diff score | 9.87 (13.65) | .36 | .13 | .98  .758 |
| Tapping contra diff score | 4.93 (13.08) | .06 | .49 | .98  .758 |
| Tapping ipsi diff score | 3.68 (12.95) | -.97 | 5.97 | .87  <.001* |
|  |  |  |  |  |
| *VAS pain pleasantness score* |  | | | |
| CT-non optimal touch contra pleasantness | 69.62 (19.03) | -.25 | -.72 | .96  .209 |
| CT-non optimal touch ips pleasantness | 71.58 (16.12) | -.46 | .12 | .96  .273 |
| CT optimal touch contra pleasantness | 75.19 (17.17) | -.30 | -1.07 | .94  .042* |
| CT optimal touch ipsi pleasantness | 71.17 (15.81) | .02 | -.54 | .97  .351 |
| Tapping contra pleasantness | 60.94 (15.57) | .15 | -.29 | .99  .972 |
| Tapping ipsi pleasantness | 58.50 (17.10) | .08 | -.52 | .98  .566 |
|  |  |  |  |  |
| BPQ | 38.08 (10.12) | -.27 | -.57 | .96  .257 |

*Significant
